# Supplementary figures and images for: The role of echinacoside-based cross-linker nanoparticles in the treatment of osteoporosis
Source: PeerJ. 2024 Apr 9;12:e17229. doi: 10.7717/peerj.17229 (PMC11011595; doi:10.7717/peerj.17229)

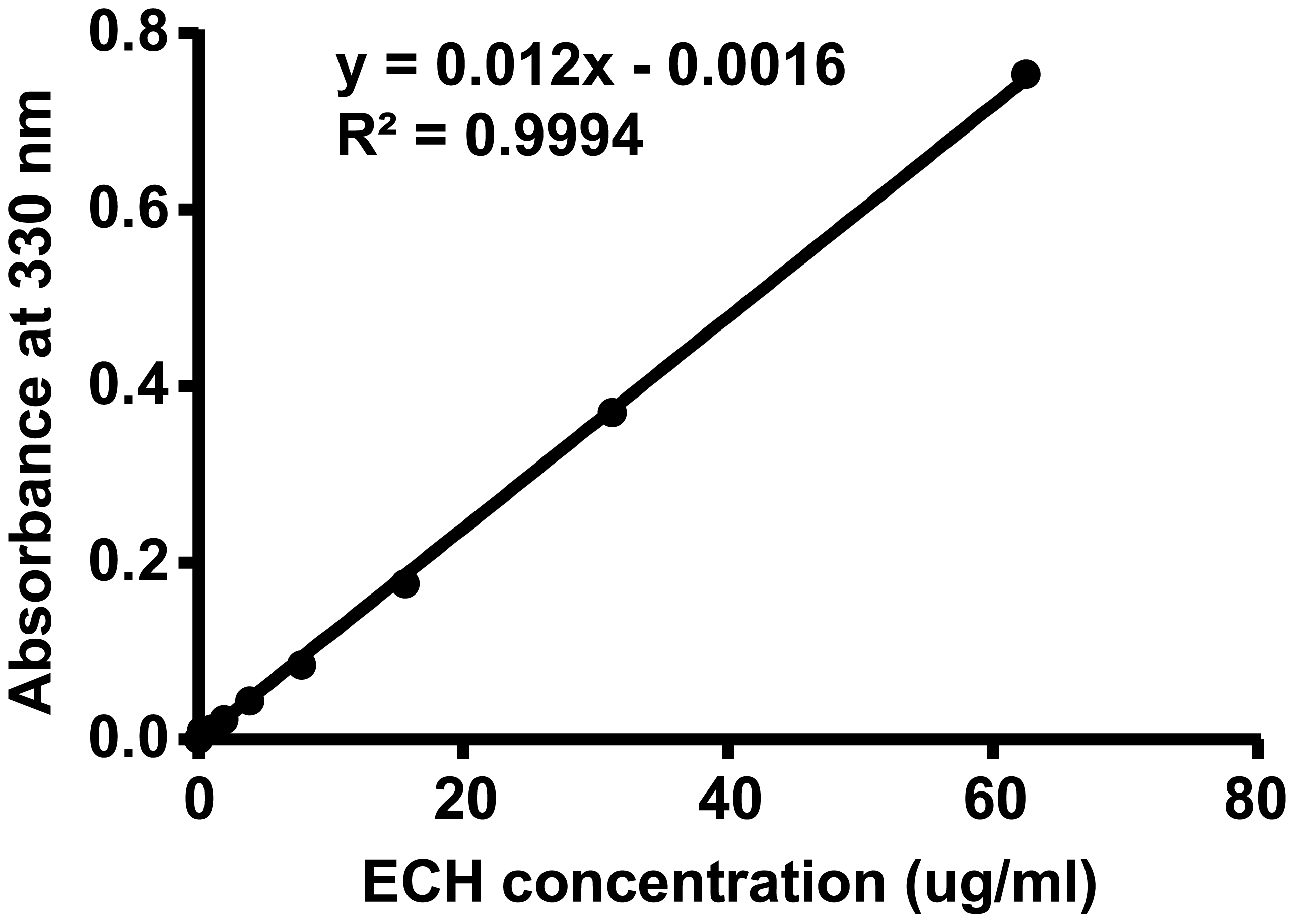

Supplement: Figure S1 [file peerj-12-17229-s001.tif]
